# A Screen for Germination Mutants in *Saccharomyces cerevisiae*

**DOI:** 10.1534/g3.111.000323

**Published:** 2011-07-01

**Authors:** Anne Kloimwieder, Fred Winston

**Affiliations:** Department of Genetics, Harvard Medical School, Boston, Massachusetts 02115

**Keywords:** *Saccharomyces cerevisiae*, germination, sporulation, *ERG6*, *TRF4*

## Abstract

Spore germination in *Saccharomyces cerevisiae* is a process in which a quiescent cell begins to divide. During germination, the cell undergoes dramatic changes in cell wall and membrane composition, as well as in gene expression. To understand germination in greater detail, we screened the *S. cerevisiae* deletion set for germination mutants. Our results identified two genes, *TRF4* and *ERG6*, that are required for normal germination on solid media. *TRF4* is a member of the TRAMP complex that, together with the exosome, degrades RNA polymerase II transcripts. *ERG6* encodes a key step in ergosterol biosynthesis. Taken together, these results demonstrate the complex nature of germination and two genes important in the process.

In the budding yeast, *Saccharomyces cerevisiae*, unfavorable growth conditions, specifically the absence of nitrogen and the presence of a nonfermentable carbon source, trigger *MAT***a***/MATα* diploid cells to undergo meiosis, forming a tetrad with four spores. During meiosis, extensive changes in transcription and histone modifications occur (Chu *et al*. 1998; Govin *et al*. 2010a; Govin *et al*. 2010b; Krishnamoorthy *et al*. 2006; Primig *et al*. 2000). Furthermore, a special spore cell wall is formed as cells go through meiosis (reviewed in Neiman 2005). Beginning in meiosis II, the prospore membrane begins to form from a collection of vesicles that flatten out to form a double membrane. The sites of prospore membrane formation are the four meiotic spindle pole bodies, which nucleate microtubules during the first meiotic division. As meiosis II proceeds, so does spore formation, as the prospore membranes expand to enclose each new haploid nucleus. After nuclear division is complete, the prospore membrane closes and spore wall synthesis begins between the two layers of the prospore membrane. The spore cell wall has two inner layers, composed mainly of mannan and beta-glucan, and two outer layers, composed mainly of chitosan and dityrosine. This is in contrast to the vegetative cell wall, which contains only two layers, composed mainly of beta-glucan and mannan. The spore cell wall, especially the two outer layers, provides protection against adverse conditions.

Spores are largely transcriptionally and translationally inert until the return to favorable growth conditions, when they undergo germination and resume vegetative growth (Brengues *et al*. 2002; Joseph-Strauss *et al*. 2007). In *S. cerevisiae* very little is known about germination, although it is clearly an important developmental process. The ras/mitogen-activated protein kinase pathway has been shown to be important for germination in *S. cerevisiae* (Herman and Rine 1997), and a recent study showed that the transcription factor Ume6 is also required for germination in *S. cerevisiae* (Strich *et al*. 2010). Germination has also been studied in other fungi, such as *Aspergillus nidulans* and *Neurospora crassa*. These studies have also implicated the ras/mitogen-activated protein kinase pathway (Osherov and May 2001; Truesdell *et al*. 1999), the cyclic AMP/protein kinase A pathway (Bruno *et al*. 1996), and the Ca^2+^/calmodulin-mediated signaling pathway (Kim *et al*. 1998; Shaw and Hoch 2000).

The transcriptional program that occurs in *S. cerevisiae* during germination can be divided into two stages: first, spores respond to glucose, and second, they respond to other nutritional components, such as amino acids (Joseph-Strauss *et al*. 2007). Gene expression during germination shares many characteristics with exit from other resting states, such as stationary phase. Like these states, germination requires large transcriptional changes in the cell, with about one-sixth of the genome undergoing transcriptional changes (Joseph-Strauss *et al*. 2007). Some of these changes in gene expression include the induction of genes associated with protein translation such as rRNA processing genes and ribosomal proteins and the repression of genes associated with the absence of an optimal carbon source such as proteasome and stress genes (Joseph-Strauss *et al*. 2007; Martinez *et al*. 2004; Radonjic *et al*. 2005).

Given the importance of germination, we wanted to identify genes required for this process. To do this, we screened the *S. cerevisiae* deletion set for germination mutants. Our results identified two genes, *TRF4* and *ERG6*. *TRF4* encodes a member of the TRAMP complex that, together with the exosome, degrades RNA polymerase II transcripts (Lacava *et al*. 2005). *ERG6* encodes a step of the ergosterol biosynthetic pathway. Ergosterol is a sterol that plays an important role in membrane fluidity (Valachovic *et al*. 2006). For both mutants, significant germination defects are observed on solid media but not in liquid media. Taken together, our results suggest that multiple functions are likely required for germination and these respond to specific environmental conditions.

## Materials and Methods

### Yeast strains and media

Except when otherwise noted, all *S. cerevisiae* strains (Table 1) are derivatives of an S288C strain with three single nucleotide polymorphisms from the SK1 background, in the genes *MKT1* and *TAO3*, and in the promoter region of *RME1*, that increase sporulation of S288C to near SK1 levels (Deutschbauer and Davis 2005). This strain background will be referred to as SK288C. Capital letters denote wild type genes, lowercase letters denote mutant alleles, and Δ indicates a complete open reading frame deletion. To create *erg6Δ::KanMX* and the other deletion alleles in SK288C background, PCR-mediated disruption of the entire open reading frame was used (Goldstein and Mccusker 1999; Sikorski and Hieter 1989). Deletions were initially made in diploids to create a heterozygote, followed by sporulation to recover haploid deletion mutants, and mating of the haploid segregants to obtain homozygous deletions. All deletions were confirmed by PCR. The SK1 alleles in the SK288C strains were confirmed by sequencing. Media and basic yeast techniques have been described previously (Rose *et al*. 1990). YPD medium (Rose *et al*. 1990) was the standard rich medium used in the germination and growth tests.

**Table 1:** *S. cerevisiae* strains used in this study

Yeast Strain	Genotype
FY4	*MAT***a**
FY2839	*MAT***a** *his3∆1 leu2∆0 lys2∆0 ura3∆0 RME1(ins-308a) TAO3(E1493Q) MKT1(D30G*)
FY2840	*MAT***a** *his3∆1 leu2∆0 lys2∆0 ura3∆0 RME1(ins-308a) TAO3(E1493Q) MKT1(D30G) trf4∆::kanMX6*
FY2841	*MATα his3∆1 leu2∆0 lys2∆0 ura3∆0 RME1(ins-308a) TAO3(E1493Q) MKT1(D30G) trf4∆::kanMX6*
FY2842	*MAT***a***/MATα RME1(ins-308a)/RME1(ins-308a) TAO3(E1493Q)/TAO3(E1493Q) MKT1(D30G)/MKT1(D30G) his3∆1/ his3∆1 leu2∆0/leu2∆0 lys2∆0/lys2∆0 ura3∆0/ura3∆0 trf4∆::kanMX6/trf4∆::kanMX6*
FY2843	*MAT***a** *his3∆1 leu2∆0 lys2∆0 ura3∆0 RME1(ins-308a) TAO3(E1493Q) MKT1(D30G) ybl083c∆::kanMX6*
FY2844	*MATα his3∆1 leu2∆0 lys2∆0 ura3∆0 RME1(ins-308a) TAO3(E1493Q) MKT1(D30G) ybl083c∆::kanMX6*
FY2845	*MAT***a** *his3∆1 leu2∆0 lys2∆0 ura3∆0 RME1(ins-308a) TAO3(E1493Q) MKT1(D30G) yml013c-a∆::kanMX6*
FY2846	*MATα his3∆1 leu2∆0 lys2∆0 ura3∆0 RME1(ins-308a) TAO3(E1493Q) MKT1(D30G) yml013c-a∆::kanMX6*
FY2847	*MAT***a***/MATα RME1(ins-308a)/RME1(ins-308a) TAO3(E1493Q)/TAO3(E1493Q) MKT1(D30G)/MKT1(D30G) his3∆1/ his3∆1 leu2∆0/leu2∆0 lys2∆0/lys2∆0 ura3∆0/ura3∆0 ybl083c∆::kanMX6/ybl083c∆::kanMX6*
FY2848	*MAT***a***/MATα RME1(ins-308a)/RME1(ins-308a) TAO3(E1493Q)/TAO3(E1493Q) MKT1(D30G)/MKT1(D30G) his3∆1/ his3∆1 leu2∆0/leu2∆0 lys2∆0/lys2∆0 ura3∆0/ura3∆0 yml013c-a∆::kanMX6/yml013c-a∆::kanMX6*
FY2853	*MATα RME1(ins-308a) TAO3(E1493Q) MKT1(D30G) erg6∆::kanMX6*
FY2854	*MAT***a** *RME1(ins-308a) TAO3(E1493Q) MKT1(D30G) erg6∆::kanMX6*
FY2855	*MATα RME1(ins-308a) TAO3(E1493Q) MKT1(D30G) htz1∆::kanMX6*
FY2856	*MAT***a** *RME1(ins-308a) TAO3(E1493Q) MKT1(D30G) htz1∆::kanMX6*
FY2857	*MAT***a***/MATα RME1(ins-308a)/RME1(ins-308a) TAO3(E1493Q)/TAO3(E1493Q) MKT1(D30G)/MKT1(D30G) erg6∆::kanMX6/erg6∆::kanMX6*
FY2858	*MAT***a***/MATα RME1(ins-308a)/RME1(ins-308a) TAO3(E1493Q)/TAO3(E1493Q) MKT1(D30G)/MKT1(D30G) htz1∆::kanMX6/htz1∆::kanMX6*

### Synthetic genetic array (SGA) screen for a germination defect by ether sensitivity

A collection of diploid yeast strains containing homozygous deletions of every nonessential gene (Giaever *et al*. 2002) was screened for defects in germination or sporulation by screening for strains unable to produce viable cells after meiosis and exposure to ether. The collection was spotted onto YPD plates, allowed to grow for 2 days at 30°C, scored for growth, and replica plated onto 1% potassium acetate sporulation plates. After 7 days, the sporulation plates were lightly replica plated to YPD and immediately treated with ether vapors. To treat cells with ether vapors, open plates were placed face down over liquid ether in a sealed container for 40 min. This treatment killed all vegetative cells, leaving only spores, which are ether resistant. Plates were then grown at room temperature for 3 days and scored for growth. Strains with wild-type growth initially and severely reduced growth after ether treatment were identified as candidate sporulation or germination mutants. The screen was performed three times. Mutants that were identified in at least two of three screens (supporting information, Table S1) were selected for further analysis.

To focus on germination, those candidates that had been previously identified as meiotic or sporulation mutants were eliminated from further analysis (Deutschbauer *et al*. 2002; Enyenihi and Saunders 2003; Marston *et al*. 2004; Rabitsch *et al*. 2001). Next, the remaining candidates were tested for their ability to sporulate. To do this, candidates were inoculated into 1 ml of YPD medium and grown to saturation overnight. The next day, 9 ml of YP-Acetate (YPA) medium was added and cultures were again grown overnight to saturation. The cultures were pelleted at 4000 rpm, washed with 10 ml dH_2_O, and then inoculated into 10 ml of 0.3% potassium acetate sporulation medium and allowed to sporulate for 7 days. After 7 days the number of tetrads was counted to assess sporulation levels. Candidates with greater than 7% tetrads in the culture were dissected to test for a potential germination defect. Candidates were determined to have a putative germination defect if there was no or little growth of tetrads after 2 days at 30°C, as compared to wild-type.

### Growth curves

Cells were inoculated and grown to saturation overnight. The next day, cells were diluted and grown to approximately 2 × 10^6^ cells/ml in YPD medium. Then, for at least four generations, cell number was determined each hour using a hemacytometer. Cells were in the logarithmic phase for the entire time course. Doubling times were calculated using linear regression with a semi-log plot.

### Budding assays

Yeast strains were first sporulated as follows. Strains were purified on YPD plates, and single colonies were inoculated into 1 ml of YPD medium and grown to saturation overnight. These cultures were then used to inoculate 10 ml of YPA medium. Cultures were again allowed to grow to saturation overnight. Cultures in YPA medium were pelleted at 4,000 rpm for 5 min at 4°C, washed with 10 ml dH_2_O, and inoculated into 10 ml of 0.3% potassium acetate sporulation medium. After 2 days, sporulation cultures were checked for tetrads. Cultures with greater than 90% sporulation were then counted and 2 × 10^7^ cells were pelleted for 2 min at 3000 rpm. The supernatant was discarded and 50 μl of 0.5 mg/ml zymolyase in 1M sorbitol was added. After 30 min at room temperature, 100 μl of 0.5% Triton-X was added and spores were mixed using a vortex. This cell suspension was then added to 2 ml of YPD to create a final cell concentration of 1 × 10^7^ cells/ml. Cells were monitored each hour for at least 6 hr. At each time point, the number of budding cells was counted as a measure of germination. At least 100 cells were counted at each time point.

### Pedigree analysis of germination

Ten microliters of sporulation culture was added to 50 µl of 0.5 mg/ml zymolyase in 1 M sorbitol and left at room temperature for 10 min. One milliliter of dH_2_O was added, and 20 µl of this cell suspension was plated in a line on a YPD plate. Wild-type spores and mutant spores were plated on the same plate directly next to one another. Ten tetrads were then dissected for each. These spores were then checked every 30 min for at least 12 hr beginning at 4 hr. The initial time when the bud could first be discerned was recorded as the time to germination. The cells were then followed for two subsequent cell divisions, again counting the time to bud as one complete cell cycle. After each cell division the cells were separated from each other by micromanipulation and moved to a new location on the plate to allow identification of the mother and daughter of each division.

### Purification of spores

Spores were purified by gradient centrifugation using a previously described method (Rockmill *et al*. 1991). For each purification, a single colony from a YPD plate was used to inoculate 10 ml of YPD liquid which was grown to saturation overnight. Each 10 ml culture was then used to inoculate 200 ml of YPA medium (10 ml into 200 ml) and this culture was grown to saturation overnight. The YPA culture was centrifuged in a Jouan CT 4 22 in a swing out rotor at 4000 rpm for 5 min at 4°C, the cell pellet was washed with 200 ml dH_2_O, and the washed cells were used to inoculate 400 ml of 0.3% potassium acetate sporulation medium. After 2 days, each sporulation culture was checked for tetrads. Cultures with greater than 90% sporulation were then centrifuged at 4000 rpm. Per gram of cells, 5 ml of 0.1 M sodium phosphate buffer pH 7.2, 2 μl concentrated mercaptoethanol, and 0.8 mg of 0.5 mg/ml zymolyase in 1 M sorbitol were added and cultures were shaken at 30°C, 190 rpm for 4 hr, dissociating tetrads into single spores. After 4 hr, 5 ml of 0.5% Triton-X were added per gram. Spores were then washed three times with 5 ml 0.5% Triton-X, pelleted each time for 5 min at 4000 rpm. After washes, the resulting pellet was resuspended in 2-3 ml of 0.5% Triton-X to generate a spore suspension of no greater than 5 ml. Percoll gradients were prepared by layering from the bottom 9 ml of each of the following four mixtures in 40 ml Sorvall tubes: (1) 8 ml Percoll, 1 ml 0.5% Triton-X, 1 ml 2.5 M sucrose; (2) 7 ml Percoll, 2 ml 0.5% Triton-X, 1 ml 2.5 M sucrose; (3) 6 ml percoll, 3 ml 0.5% Triton-X, 1 ml 2.5 M sucrose; and (4) 5 ml Percoll, 4 ml 0.5% Triton-X, 1 ml 2.5 M sucrose. On top of this gradient, 1.5 ml of the spore suspension was layered. These gradients were then spun at 10,000 rpm at 4°C for 1 hr in a SA-600 rotor in a Sorvall centrifuge. After centrifugation, the top three layers, consisting of vegetative cells and debris were removed and discarded. The remaining spore layer, consisting of >99.9% spores, was then removed by pipette and washed three times with 30 ml 0.5% Triton-X. After the washes, spores were re-suspended in 5 ml 0.5% Triton-X and stored at 4°C. Percoll was purchased from MP Biomedical.

### Microscopy time courses

Purified spores were diluted to between 1-5 × 10^7^ cells/ml and sonicated to disperse the spores. Two small agar pads were created by dissolving 0.1 g of low-melt agarose into 5 ml SC-complete medium and plating approximately 1 ml sandwiched between two cover slips. Then, 0.5 μl of wild-type or deletion mutant cells were spread on separate agar pads. These agar pads were then placed cell side down on a glass slide so that the spores were sandwiched between the glass slide and the agar. The glass slide was contained in a small dish to prevent the agar from drying out. Cells were then monitored on a Nikon TE2000 microscope with Perfect Focus, 100× NA 1.4 objective at 30°C for at least 12 hr by image capture every 5 min. After image capture cells were individually tracked for germination by the appearance of the first bud. Cells were also monitored for two subsequent cell divisions by bud appearance.

## Results

### A screen for *S. cerevisiae* germination mutants

**Figure 1:**
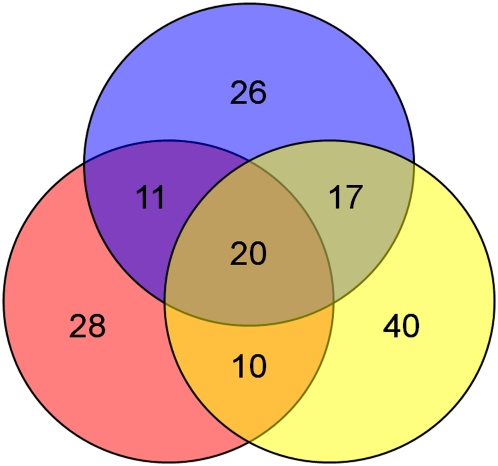
Results from three screens for germination mutants. A Venn diagram shows results from the three screens for germination mutants.

To identify genes required for germination, we performed a screen of the *S. cerevisiae* diploid deletion set to identify mutants defective for this process (described in *Materials and Methods*). Because our screen would identify both meiosis and germination mutants, we removed from consideration any meiosis or sporulation mutants identified in previous screens (Deutschbauer *et al*. 2002; Enyenihi and Saunders 2003; Marston *et al*. 2004; Rabitsch *et al*. 2001). Our screen was performed three times, each time identifying an average of 77 candidates, with an overlap of approximately 56% with at least one of the other two screens. The 58 mutants identified in at least two of the three screens were included in secondary analysis (Figure 1).

Each of the 58 candidates was tested for sporulation and germination. Of these, twelve mutants sporulated and produced complete tetrads at a sufficient level (above 7% sporulation) to analyze possible germination defects (Table 2). Four of the twelve mutants appeared to have a germination defect: *trf4Δ*, *erg6Δ*, *ybl083cΔ*, and *yml013c-aΔ*. Of the mutants that exhibited a sporulation defect, several seemed to be functionally related in mRNA export, Cdc48 function, or ESCRTIII complex.

**Table 2:** Sporulation in diploid deletion strains

Gene	Percent Sporulation	Gene	Percent Sporulation
*YEL045C*	27.6	*RMD7*	0.0
*PHO88*	20.7	*RVS161*	0.0
*BUD30*	18.9	*SEC22*	0.0
*YBL083C*	18.0	*VPS4*	0.0
*SNT309*	17.7	*PFK1*	0.0
*YML013C-A*	16.5	*NEM1*	0.0
*ERG6*	10.0	*KCS1*	0.0
*NEW1*	9.8	*POP2*	0.0
*LHS1*	8.9	*VAM6*	0.0
*UBP6*	7.8	*YGR162W*	0.0
*RPA49*	7.6	*SPT20*	0.0
*TRF4*	7.2	*SLG1*	0.0
*SEL1*	6.6	*SSE1*	0.0
*BRR1*	5.9	*NPR2*	0.0
*PRO1*	4.5	*YKL118W*	0.0
*IWR1*	2.7	*VPH2*	0.0
*YDR433W*	2.0	*SFP1*	0.0
*DID2*	1.6	*YLR235C*	0.0
*PAT1*	1.4	*RAI1*	0.0
*YNL025C*	1.4	*BRO1*	0.0
*YKR035C*	1.4	*RAD6*	0.0
*PLC1*	1.3	*DOA1*	0.0
*YME1*	0.8	*VPS20*	0.0
*RPL22A*	0.5	*STO1*	0.0
*CBC2*	0.5	*YNR042W*	0.0
*PHO86*	0.4	*PET494*	0.0
*SNF7*	0.4	*IMP2*'	0.0
*MET22*	0.0	*SLX8*	0.0
*IES6*	0.0	*VAM7*	0.0
*VMA8*	0.0	*SHP1*	0.0

To study the germination phenotype of each deletion in a genetic background in which sporulation occurs at a high frequency, each deletion was constructed in the SK288C strain background (see *Materials and Methods*). In this strain background, two of the candidates, *trf4∆* and *erg6∆*, showed germination defects after dissection of tetrads on rich (YPD) plates. *TRF4* encodes a member of the TRAMP complex, that together with the exosome, degrades RNA polymerase II transcripts (Wyers *et al*. 2005). *ERG6* encodes a step of the ergosterol biosynthetic pathway (Gaber *et al*. 1989).

### Analysis of germination in *trf4Δ* mutants

**Figure 2:**
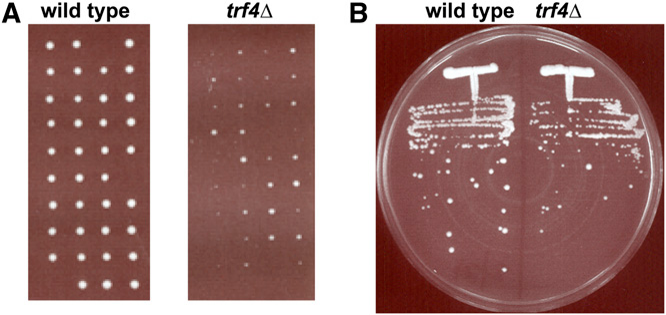
Growth of *trf4Δ* mutants on solid media. (A) Wild-type and homozygous *trf4*Δ diploids were sporulated and dissected on YPD plates. Shown are the germination plates, each after 2 d of incubation at 30°C. (B) Wild-type and *trf4Δ* haploid strains were streaked for single colonies on YPD medium and were incubated for 2 d at 30°C.

To assess the *trf4Δ* germination phenotype, both homozygous wild-type and *trf4Δ/trf4Δ* diploids were sporulated and tetrads were dissected on YPD plates. Germination was assessed by comparing the growth of the wild-type and *trf4Δ* strains after germination and after normal vegetative growth. As can be seen in Figure 2, *trf4Δ* spores exhibit a growth defect compared with wild-type (Figure 2A). When vegetatively growing cells are grown on a YPD plate, the wild-type and *trf4Δ* strains grow at a more comparable level, although there is a mild growth impairment for *trf4Δ* (Figure 2B). The more severe growth defect after germination indicates that *trf4∆* cells have a germination defect on solid media.

**Figure 3:**
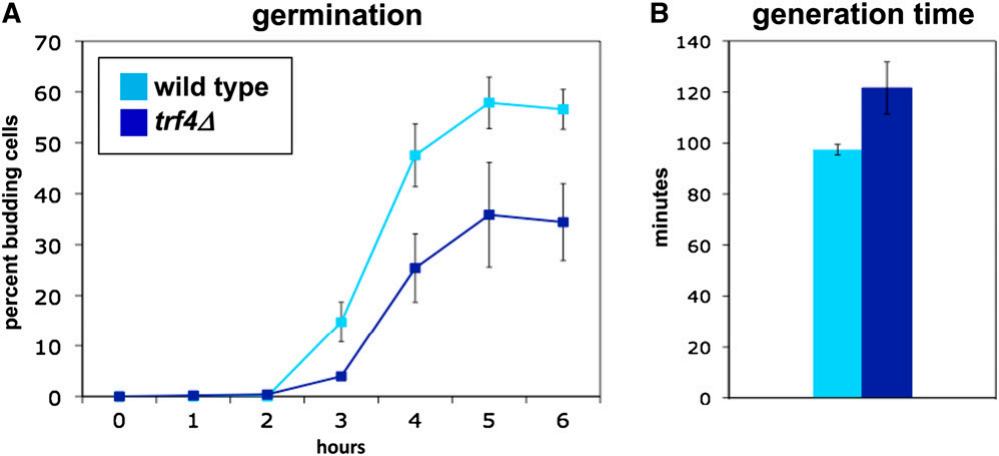
Analysis of *trf4Δ* germination and growth in liquid media. (A) Wild-type and *trf4∆* spores were inoculated into YPD liquid media. The number of budding cells was counted each hour as a measure of germination. Shown here is the average ± SE of five experiments. (B) Wild-type and *trf4∆* strains in logarithmic phase were counted every hour to determine their generation times in liquid media. Shown is the average ± SE of three experiments.

To assay the *trf4Δ* germination defect in a more quantitative fashion, germination time courses were performed in liquid YPD medium. To do this, purified spores were inoculated into liquid YPD medium and monitored over a time course of six hours. At each hour, the number of budding cells was counted as a measure of germination. Our results (Figure 3A) show that, in liquid YPD medium, germination occurred asynchronously, over approximately 3 hr for both wild-type and *trf4Δ* spores. Furthermore, the kinetics of appearance of budded cells was similar between the two strains, although the number of germinating *trf4Δ* spores was less than the number for wild-type throughout the time course, suggesting that fewer *trf4Δ* spores were able to germinate. We also measured the generation time for wild-type and *trf4∆* mutants growing vegetatively in YPD liquid medium and found that the *trf4Δ* mutant had a modest growth defect (Figure 3B). From these results, we are unable to conclude that *trf4Δ* germination in liquid YPD medium is slower; however, *trf4Δ* does cause a decrease in the frequency of spores that can germinate in both liquid and solid YPD.

**Figure 4:**
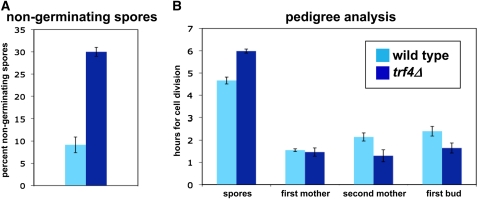
Pedigree analysis of germination in a *trf4Δ* mutant. (A) Percent of nongerminating spores after tetrad analysis of wild-type and *trf4Δ/trf4Δ* diploids. Shown are the average times ± SE for three experiments. (B) Time to complete germination and initial cell divisions on solid media. Shown are the average times ± SE for three experiments for germination and early cell divisions after germination. The times indicate the first detectable appearance of a bud.

To further analyze *trf4Δ*, pedigree analysis was performed to measure germination time on solid medium. In this analysis, spores were dissected to specific positions on a YPD plate. Each spore was then monitored for the time to germinate. In addition, two additional rounds of cell division were monitored. As shown in Figure 4, *trf4Δ* spores germinate more slowly than wild-type on YPD plates. First, compared with wild-type, many spores do not germinate at all (Figure 4A). Among the cells that do germinate, the *trf4Δ* mutants averaged over an hour longer than wild-type (6 hr *vs*. 4.7 hr; Figure 4B). In contrast to the longer time for *trf4Δ* spores to germinate, there was no *trf4Δ* growth defect observed in the subsequent generations that were monitored (Figure 4B). In fact, the *trf4Δ* cells appeared to divide slightly faster than wild-type. In conclusion, on YPD plates, *trf4Δ* mutants appear to germinate at lower frequency and more slowly than wild-type.

### Analysis of *erg6Δ*

**Figure 5:**
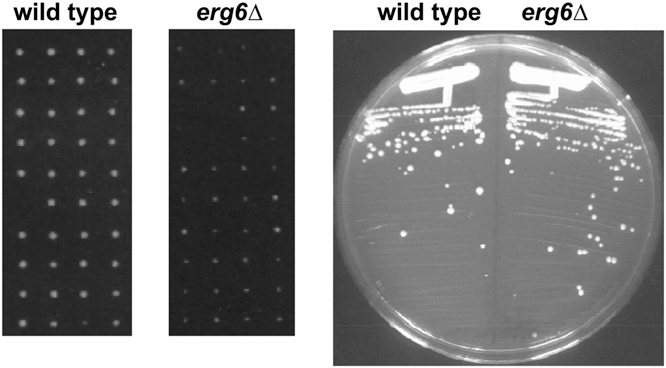
Growth of *erg6Δ* mutants on solid media. (A) Wild-type and homozygous *erg6*Δ diploids were sporulated and dissected on YPD plates. Shown are the germination plates, each after 2 d of incubation at 30°C. (B) Wild-type and *erg6Δ* haploid strains were streaked for single colonies on YPD medium and were incubated for 2 d at 30°C.

The second mutant that showed a germination defect was *erg6Δ*. Because previous studies have suggested that particular auxotrophies could affect the growth of *erg6Δ* mutants (Boer *et al*. 2008; Gaber *et al*. 1989), all *erg6Δ* experiments were done with prototrophic strains. To examine the effect of an *erg6Δ* mutation on germination, an SK288C homozygous *erg6Δ* diploid was sporulated and tetrads were dissected. Our results show that *erg6Δ* spores exhibit a severe growth defect after dissection on YPD plates compared to a wild-type diploid (Figure 5A). Based on colony size, this defect is unlikely to be caused by poorer vegetative growth (Figure 5B).

**Figure 6:**
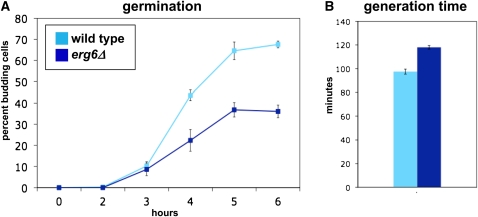
Analysis of *erg6Δ* germination and growth in liquid media. (A) Wild-type and *erg6∆* spores were inoculated into YPD liquid media. The number of budding cells was counted each hour as a measure of germination. Shown here is the average of ± SE of three experiments. (B) Wild-type and *erg6∆* strains in logarithmic phase were counted every hour to determine their generation times time in liquid media. Shown is the average ± SE of three measurements.

To examine the effect of *erg6Δ* on germination in liquid YPD medium, time courses were performed using purified spores, monitoring germination by the timing of bud emergence. As shown in Figure 6A, *erg6Δ* spores exhibited a defect in germination, both in terms of the rate at which buds appeared and the percentage of spores that budded. To determine whether the germination defect might be related to slower growth of *erg6Δ* mutants, the generation time in liquid YPD was measured. Our results show that *erg6Δ* mutants do have a longer generation time in liquid medium (Figure 6B). The slower growth of *erg6Δ* in liquid YPD might account for some of the differences seen for the rate of germination, although it would not account for the lower frequency of spores that are able to germinate.

**Figure 7:**
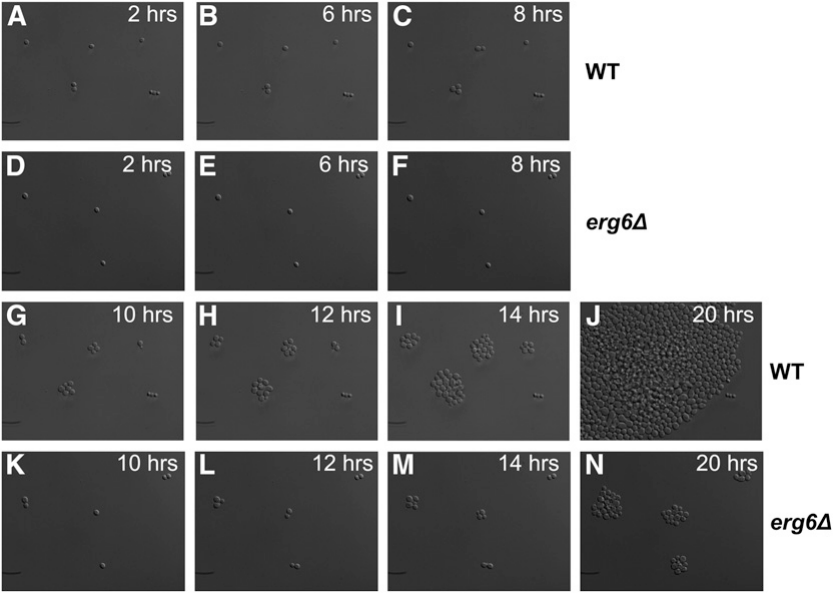
Live cell imaging. Sample frames from live cell imaging of wild-type (A–C, G–J) and *erg6Δ* (D–F, K–N) cells. Time points are as follows: A and D, 2 hr; B and E, 6 hr; C and F, 8 hr; G and K, 10 hr; H and L, 12 hr; I and M, 14 hr; and J and N, 20 hr and 20 min.

To gain an additional view of the germination defect in *erg6Δ* mutants, individual spores on SC agar were used for live cell imaging, with pictures taken every 5 min at multiple positions for both the wild-type and *erg6Δ* spores. Sample frames are shown in Figure 7, while a movie is shown as Figure S1. Using these images, each spore was monitored for the time to germinate. From this analysis, *erg6Δ* spores averaged a time to bud of 7.4 ± 0.77 hr, while wild-type averaged 5.4 ± 0.77 hr, a statistically significant difference (*P* = 0.0019) and greater than the vegetative growth difference as judged by colony size (Figure 5B) or by measurement of generation time (Figure 6B). Taken together, these analyses show that *erg6∆* mutants exhibit a germination defect on solid media.

## Discussion

In this study, we screened for *S. cerevisiae* germination mutants, leading to the identification of two genes, *TRF4* and *ERG6*. Both *trf4∆* and *erg6∆* mutants exhibit germination defects on solid media, while the results are less clear in liquid media. On solid media, the *trf4Δ* mutants had a lower percentage of spores able to germinate, and those that did, took significantly longer. While the *erg6Δ* mutant spores germinated at a frequency close to that of wild-type, they took longer and, interestingly, exhibited slower growth for at least the first two cell divisions after germination. A previous screen for germination mutants also identified *trf4∆* mutants, although this screen was done in liquid media (Deutschbauer *et al*. 2002).

There are several reasons why *TRF4* might be important for germination. Trf4 is a member of the TRAMP complex, which degrades cryptic unstable transcripts (CUTs) (Butler 2002; Davis and Ares 2006; Wyers *et al*. 2005). The TRAMP complex also plays a role in degrading antisense transcripts (Camblong *et al*. 2007), in stimulating the degradation of spliced out introns, and in telomere maintenance (San Paolo *et al*. 2009). Additionally, the TRAMP complex has been shown to play a role in regulating histone levels (Reis and Campbell 2007). Thus, a *trf4Δ* mutation might indirectly impair germination by alterations in gene expression, including an alteration in histone levels.

The role of Erg6 in maintaining proper cell membrane composition could be responsible for the germination defects seen in *erg6∆* mutants. In the absence of Erg6, which catalyzes a step in ergosterol biosynthesis, cells produce zymosterol instead of ergosterol, changing the composition of the cell membrane (Bard *et al*. 1977). These changes result in changes in membrane fluidity and could result in changes in lipid rafts, which are important for cell signaling (Gaber *et al*. 1989; Sharma 2006; Valachovic *et al*. 2006). Changes in cell signaling could play an important role in spore germination leading to the delayed germination seen in *erg6∆* mutants. We were unable to test whether the addition of exogenous ergosterol is capable of rescuing the observed defect, as *S. cerevisiae* is unable to take up ergosterol under aerobic conditions (Lewis *et al*. 1985).

One intriguing result is that the germination defects for both *trf4Δ* and *erg6Δ* are more pronounced on solid than liquid media. Cells, and specifically spores, have been previously observed to behave differently on solid *vs*. liquid media (Piccirillo *et al*. 2010). Differences on solid media *vs*. liquid media is also the most probable reason that our screen failed to identify previously identified germination mutants as our screen was conducted on solid media while previous screens were conducted in liquid media (Deutschbauer *et al*. 2002; Herman and Rine 1997). Taken together, the results described here present evidence for two genes, *TRF4* and *ERG6*, with roles in germination.

## Supplementary Material

Supporting Information

## References

[bib1] Bard M. Woods R. A. Barton D. H. Corrie J. E. Widdowson D. A., 1977 Sterol mutants of *Saccharomyces cerevisiae*: chromatographic analyses. Lipids 12: 645–654 331007 10.1007/BF02533759

[bib2] Boer V. M. Amini S. Botstein D., 2008 Influence of genotype and nutrition on survival and metabolism of starving yeast. Proc. Natl. Acad. Sci. USA 105: 6930–6935 18456835 10.1073/pnas.0802601105 PMC2383937

[bib3] Brengues M. Pintard L. Lapeyre B., 2002 mRNA decay is rapidly induced after spore germination of *Saccharomyces cerevisiae*. J. Biol. Chem. 277: 40505–40512 12181322 10.1074/jbc.M206700200

[bib4] Bruno K. S. Aramayo R. Minke P. F. Metzenberg R. L. Plamann M., 1996 Loss of growth polarity and mislocalization of septa in a Neurospora mutant altered in the regulatory subunit of cAMP-dependent protein kinase. EMBO J. 15: 5772–5782 8918454 PMC452324

[bib5] Butler J. S., 2002 The yin and yang of the exosome. Trends Cell Biol. 12: 90–96 11849973 10.1016/s0962-8924(01)02225-5

[bib6] Camblong J. Iglesias N. Fickentscher C. Dieppois G. Stutz F., 2007 Antisense RNA stabilization induces transcriptional gene silencing via histone deacetylation in *S. cerevisiae*. Cell 131: 706–717 18022365 10.1016/j.cell.2007.09.014

[bib7] Chu S. DeRisi J. Eisen M. Mulholland J. Botstein D., 1998 The transcriptional program of sporulation in budding yeast. Science 282: 699–705 9784122 10.1126/science.282.5389.699

[bib8] Davis C. A. Ares M. Jr, 2006 Accumulation of unstable promoter-associated transcripts upon loss of the nuclear exosome subunit Rrp6p in *Saccharomyces cerevisiae*. Proc. Natl. Acad. Sci. USA 103: 3262–3267 16484372 10.1073/pnas.0507783103 PMC1413877

[bib9] Deutschbauer A. M. Davis R. W., 2005 Quantitative trait loci mapped to single-nucleotide resolution in yeast. Nat. Genet. 37: 1333–1340 16273108 10.1038/ng1674

[bib10] Deutschbauer A. M. Williams R. M. Chu A. M. Davis R. W., 2002 Parallel phenotypic analysis of sporulation and postgermination growth in *Saccharomyces cerevisiae*. Proc. Natl. Acad. Sci. USA 99: 15530–15535 12432101 10.1073/pnas.202604399 PMC137751

[bib11] Enyenihi A. H. Saunders W. S., 2003 Large-scale functional genomic analysis of sporulation and meiosis in *Saccharomyces cerevisiae*. Genetics 163: 47–54 12586695 10.1093/genetics/163.1.47 PMC1462418

[bib12] Gaber R. F. Copple D. M. Kennedy B. K. Vidal M. Bard M., 1989 The yeast gene ERG6 is required for normal membrane function but is not essential for biosynthesis of the cell-cycle-sparking sterol. Mol. Cell. Biol. 9: 3447–3456 2677674 10.1128/mcb.9.8.3447-3456.1989 PMC362391

[bib13] Giaever G. Chu A. M. Ni L. Connelly C. Riles L., 2002 Functional profiling of the *Saccharomyces cerevisiae* genome. Nature 418: 387–391 12140549 10.1038/nature00935

[bib14] Goldstein A. L. McCusker J. H., 1999 Three new dominant drug resistance cassettes for gene disruption in *Saccharomyces cerevisiae*. Yeast 15: 1541–1553 10514571 10.1002/(SICI)1097-0061(199910)15:14<1541::AID-YEA476>3.0.CO;2-K

[bib15] Govin J. Dorsey J. Gaucher J. Rousseaux S. Khochbin S., 2010a Systematic screen reveals new functional dynamics of histones H3 and H4 during gametogenesis. Genes Dev. 24: 1772–1786 20713519 10.1101/gad.1954910 PMC2922505

[bib16] Govin J. Schug J. Krishnamoorthy T. Dorsey J. Khochbin S., 2010b Genome-wide mapping of histone H4 serine-1 phosphorylation during sporulation in *Saccharomyces cerevisiae*. Nucleic Acids Res. 38: 4599–4606 20375100 10.1093/nar/gkq218 PMC2919718

[bib17] Herman P. K. Rine J., 1997 Yeast spore germination: a requirement for Ras protein activity during re-entry into the cell cycle. EMBO J. 16: 6171–6181 9321396 10.1093/emboj/16.20.6171 PMC1326301

[bib18] Joseph-Strauss D. Zenvirth D. Simchen G. Barkai N., 2007 Spore germination in *Saccharomyces cerevisiae*: global gene expression patterns and cell cycle landmarks. Genome Biol. 8: R241 17999778 10.1186/gb-2007-8-11-r241 PMC2258198

[bib19] Kim Y. K. Li D. Kolattukudy P. E., 1998 Induction of Ca2+-calmodulin signaling by hard-surface contact primes Colletotrichum gloeosporioides conidia to germinate and form appressoria. J. Bacteriol. 180: 5144–5150 9748448 10.1128/jb.180.19.5144-5150.1998 PMC107551

[bib20] Krishnamoorthy T. Chen X. Govin J. Cheung W. L. Dorsey J., 2006 Phosphorylation of histone H4 Ser1 regulates sporulation in yeast and is conserved in fly and mouse spermatogenesis. Genes Dev. 20: 2580–2592 16980586 10.1101/gad.1457006 PMC1578680

[bib21] LaCava J. Houseley J. Saveanu C. Petfalski E. Thompson E., 2005 RNA degradation by the exosome is promoted by a nuclear polyadenylation complex. Cell 121: 713–724 15935758 10.1016/j.cell.2005.04.029

[bib22] Lewis T. A. Taylor F. R. Parks L. W., 1985 Involvement of heme biosynthesis in control of sterol uptake by *Saccharomyces cerevisiae*. J. Bacteriol. 163: 199–207 3891725 10.1128/jb.163.1.199-207.1985 PMC219098

[bib23] Marston A. L. Tham W. H. Shah H. Amon A., 2004 A genome-wide screen identifies genes required for centromeric cohesion. Science 303: 1367–1370 14752166 10.1126/science.1094220

[bib24] Martinez M. J. Roy S. Archuletta A. B. Wentzell P. D. Anna-Arriola S. S., 2004 Genomic analysis of stationary-phase and exit in *Saccharomyces cerevisiae*: gene expression and identification of novel essential genes. Mol. Biol. Cell 15: 5295–5305 15456898 10.1091/mbc.E03-11-0856 PMC532011

[bib25] Neiman A. M., 2005 Ascospore formation in the yeast *Saccharomyces cerevisiae*. Microbiol. Mol. Biol. Rev. 69: 565–584 16339736 10.1128/MMBR.69.4.565-584.2005 PMC1306807

[bib26] Osherov N. May G. S., 2001 The molecular mechanisms of conidial germination. FEMS Microbiol. Lett. 199: 153–160 11377860 10.1111/j.1574-6968.2001.tb10667.x

[bib27] Piccirillo S. White M. G. Murphy J. C. Law D. J. Honigberg S. M., 2010 The Rim101p/PacC pathway and alkaline pH regulate pattern formation in yeast colonies. Genetics 184: 707–716 20038633 10.1534/genetics.109.113480 PMC2845339

[bib28] Primig M. Williams R. M. Winzeler E. A. Tevzadze G. G. Conway A. R., 2000 The core meiotic transcriptome in budding yeasts. Nat. Genet. 26: 415–423 11101837 10.1038/82539

[bib29] Rabitsch K. P. Toth A. Galova M. Schleiffer A. Schaffner G., 2001 A screen for genes required for meiosis and spore formation based on whole-genome expression. Curr. Biol. 11: 1001–1009 11470404 10.1016/s0960-9822(01)00274-3

[bib30] Radonjic M. Andrau J. C. Lijnzaad P. Kemmeren P. Kockelkorn T. T., 2005 Genome-wide analyses reveal RNA polymerase II located upstream of genes poised for rapid response upon *S. cerevisiae* stationary phase exit. Mol. Cell 18: 171–183 15837421 10.1016/j.molcel.2005.03.010

[bib31] Reis C. C. Campbell J. L., 2007 Contribution of Trf4/5 and the nuclear exosome to genome stability through regulation of histone mRNA levels in *Saccharomyces cerevisiae*. Genetics 175: 993–1010 17179095 10.1534/genetics.106.065987 PMC1840065

[bib32] Rockmill B. Lambie E. J. Roeder G. S., 1991 Spore enrichment. Methods Enzymol. 194: 146–149 2005784 10.1016/0076-6879(91)94012-2

[bib33] Rose M. D. Winston F. Hieter P., 1990 Methods in Yeast Genetics: A Laboratory Course Manual: New York. Cold Spring Harbor Laboratory Press, Cold Spring Harbor, NY

[bib34] San Paolo S. Vanacova S. Schenk L. Scherrer T. Blank D., 2009 Distinct roles of non-canonical poly(A) polymerases in RNA metabolism. PLoS Genet. 5: e1000555 19593367 10.1371/journal.pgen.1000555 PMC2700272

[bib35] Sharma S. C., 2006 Implications of sterol structure for membrane lipid composition, fluidity and phospholipid asymmetry in *Saccharomyces cerevisiae*. FEM. Yeast Res. 6: 1047–1051 10.1111/j.1567-1364.2006.00149.x 17042754

[bib36] Shaw B. D. Hoch H. C., 2000 Ca2+ regulation of Phyllosticta ampelicida pycnidiospore germination and appressorium Formation. Fungal Genet. Biol. 31: 43–53 11118134 10.1006/fgbi.2000.1223

[bib37] Sikorski R. S. Hieter P., 1989 A system of shuttle vectors and yeast host strains designed for efficient manipulation of DNA in *Saccharomyces cerevisiae*. Genetics 122: 19–27 2659436 10.1093/genetics/122.1.19 PMC1203683

[bib38] Strich R. Khakhina S. Mallory M. J., 2011 Ume6p is required for germination and early colony development of yeast ascospores. FEMS Yeast Res. 11: 104–113 21059190 10.1111/j.1567-1364.2010.00696.x PMC3951096

[bib39] Truesdell G. M. Jones C. Holt T. Henderson G. Dickman M. B., 1999 A Ras protein from a phytopathogenic fungus causes defects in hyphal growth polarity, and induces tumors in mice. Mol. Gen. Genet. 262: 46–54 10503535 10.1007/s004380051058

[bib40] Valachovic M. Bareither B. M. Shah Alam Bhuiyan M. Eckstein J. Barbuch R., 2006 Cumulative mutations affecting sterol biosynthesis in the yeast *Saccharomyces cerevisiae* result in synthetic lethality that is suppressed by alterations in sphingolipid profiles. Genetics 173: 1893–1908 16702413 10.1534/genetics.105.053025 PMC1569731

[bib41] Wyers F. Rougemaille M. Badis G. Rousselle J. C. Dufour M. E., 2005 Cryptic pol II transcripts are degraded by a nuclear quality control pathway involving a new poly(A) polymerase. Cell 121: 725–737 15935759 10.1016/j.cell.2005.04.030

