# Supplementary material for: A Screen for Germination Mutants in Saccharomyces cerevisiae
Source: G3 (Bethesda). 2011 Jul 1;1(2):143–9. doi: 10.1534/g3.111.000323 (PMC3276131; doi:10.1534/g3.111.000323)
Supplement: Supporting Information [file supp_1_2_143__index.html]

Supporting Information 

# A Screen for Germination Mutants in *Saccharomyces cerevisiae*

## Supporting Information for Kloimwieder and Winston, 2011

**Files in this Data Supplement:**

- Supporting Information - Figure S1 and Table S1 (PDF, 68 KB)
- Table S1 - Genes that affect either sensitivity (PDF, 40 KB)
- Figure S1 - Microscopy time course of germination (.mov, 812 KB)
