# Supplementary material for: A Screen for Germination Mutants in Saccharomyces cerevisiae
Source: G3 (Bethesda). 2011 Jul 1;1(2):143–9. doi: 10.1534/g3.111.000323 (PMC3276131; doi:10.1534/g3.111.000323)
Supplement: Supporting Information [file supp_1.2.143_000323SI.pdf]

Figure S1 Microscopy time course of germination. Spores were prepared as described in Materials and Methods. Shown are images taken every five minutes. The left panel shows spores from a wild-type strain and the right panel shows spores from an *erg6Δ* mutant.

Figure S1 is available for download as a movie file at http://www.g3journal.org/lookup/suppl/doi:10.1534/g3.111.000323/-/DC1/FigureS1.mov.

Table S1 Genes that affect either sensitivity

Systematic name	Common name	Systematic name	Common name	Systematic name	Common name
YAL026C	DRS2	YGL084C	GUP1	YML013C-A	
YAL056W	GPB2	YGL212W	VAM7	YML013W	SEL1
YBL058W	SHP1	YGL246C	RAI1	YML014W	TRM9
YBL083C		YGR162W		YMR077C	VPS20
YBR106W		YGR240C	PFK1	YMR125W	STO1
YBR126C		YGR252W		YNL025C	
YBR283C	SSH1	YHL023C	RMD11	YNL117W	
YCR009C	RVS161	YHR004C	NEM1	YNL220W	
YCR094W	CDC50	YHR116W	COX23	YNL248C	RPA49
YDL077C		YHR194W	MDM31	YNR006W	
YDL115C	IWR1	YIL154C		YNR041C	
YDL151C	BUD30	YJL117W	PHO86	YNR042W	
YDR017C	KCS1	YJL188C	BUD19	YNR045W	
YDR207C	UME6	YJR095W	SFC1	YNR052C	
YDR264C	AKR1	YKL041W	VPS24	YOL064C	MET22
YDR300C	PRO1	YKL057C	NUP120	YOL071W	EMI5
YDR378C	LSM6	YKL073W	LHS1	YOL115W	TRF4
YDR388W	RVS167	YKL118W		YOL148C	
YDR433W		YKL119C	VPH2	YOR008C	SLG1
YDR477W		YKL213C	DOA1	YOR014W	RTS1
YEL044W	IES6	YKR035C		YOR080W	DIA2
YEL045C		YKR097W	PCK1	YPL084W	BRO1
YEL046C	GLY1	YLL039C	UBI4	YPL106C	SSE1
YEL051W	VMA8	YLR025W	SNF7	YPL178W	CBC2
YEL060C	PRB1	YLR052W	IES3	YPL205C	
YEL062W	NPR2	YLR061W	RPL22A	YPL226W	NEW1
YER065C	ICL1	YLR087C	CSF1	YPL268W	
YER083C	RMD7	YLR268W	SEC22	YPR024W	
YER116C		YLR270W	DCS1	YPR057W	BRR1
YFL033C		YLR377C	FBP1	YPR101W	SNT309
YFR010W	UBP6	YLR403W	SFP1	YPR139C	VPS66
YGL058W	RAD6	YML001W	YPT7	YPR173C	VPS4
		YML008C	ERG6		
